# NeuroVI-based wave compensation system control for offshore wind turbines

**DOI:** 10.3389/fnbot.2025.1648713

**Published:** 2025-07-30

**Authors:** Fengshuang Ma, Xiangyong Liu, Zhiqiang Xu, Tianhong Ding

**Affiliations:** ^1^Fishery Machinery and Instrument Research Institute, Chinese Academy of Fishery Sciences, Shanghai, China; ^2^East China Sea Fisheries Research Institute, Chinese Academy of Fishery Sciences, Shanghai, China

**Keywords:** integrated installation of offshore wind turbine, wave compensation system, neuromorphic vision (NeuroVI) camera, spiking neural networks (SNN), AMESim-Simulink co-simulation, synchronized motion control of displacements

## Abstract

In deep-sea areas, the hoisting operation of offshore wind turbines is seriously affected by waves, and the secondary impact is prone to occur between the turbine and the pile foundation. To address this issue, this study proposes an integrated wave compensation system for offshore wind turbines based on a neuromorphic vision (NeuroVI) camera. The system employs a NeuroVI camera to achieve non-contact, high-precision, and low-latency displacement detection of hydraulic cylinders, overcoming the limitations of traditional magnetostrictive displacement sensors, which exhibit slow response and susceptibility to interference in harsh marine conditions. A dynamic simulation model was developed using AMESim-Simulink co-simulation to analyze the compensation performance of the NeuroVI-based system under step and sinusoidal wave disturbances. Comparative results demonstrate that the NeuroVI feedback system achieves faster response times and superior stability over conventional sensors. Laboratory-scale model tests and real-world application in the installation of a 5.2 MW offshore wind turbine validated the system’s feasibility and robustness, enabling real-time collaborative control of turbine and cylinder displacement to effectively mitigate multi-impact risks. This research provides an innovative approach for deploying neural perception technology in complex marine scenarios and advances the development of neuro-robotic systems in ocean engineering.

## 1 Introduction

With the rapid expansion of offshore wind power development, deep-sea areas are progressively becoming the primary focus of future projects (Zhang J. et al., 2024; Yi, 2025). However, significant wave disturbances in deep-sea environments frequently cause multiple collisions between wind turbines and pile foundations during integrated installation processes, potentially leading to structural damage (Liu Y. et al., 2022). Wave compensation systems (Wu et al., 2024; Bai and Hu, 2016) actively or passively counteract irregular motions of mother vessels or offshore equipment induced by wave action. By enabling real-time dynamic position adjustments, these systems mitigate wave-induced movements, ensuring safe and reliable offshore operations (Halvorsen et al., 2021; Elbadawy and Shehata, 2015). Consequently, designing high-response, high-stability wave compensation systems is crucial for securing installation safety.

During the integrated installation process of offshore wind turbines, the crane vessel is subjected to six-degree-of-freedom (6-DoF) wave-induced motions, including surge, sway, heave, roll, pitch, and yaw (Wang, 2021). Given that the total weight of a wind turbine typically exceeds a thousand tons, heave motion has the most significant impact on lifting stability. Therefore, an active wave compensation system is essential for motion suppression.

**Figure 1:**
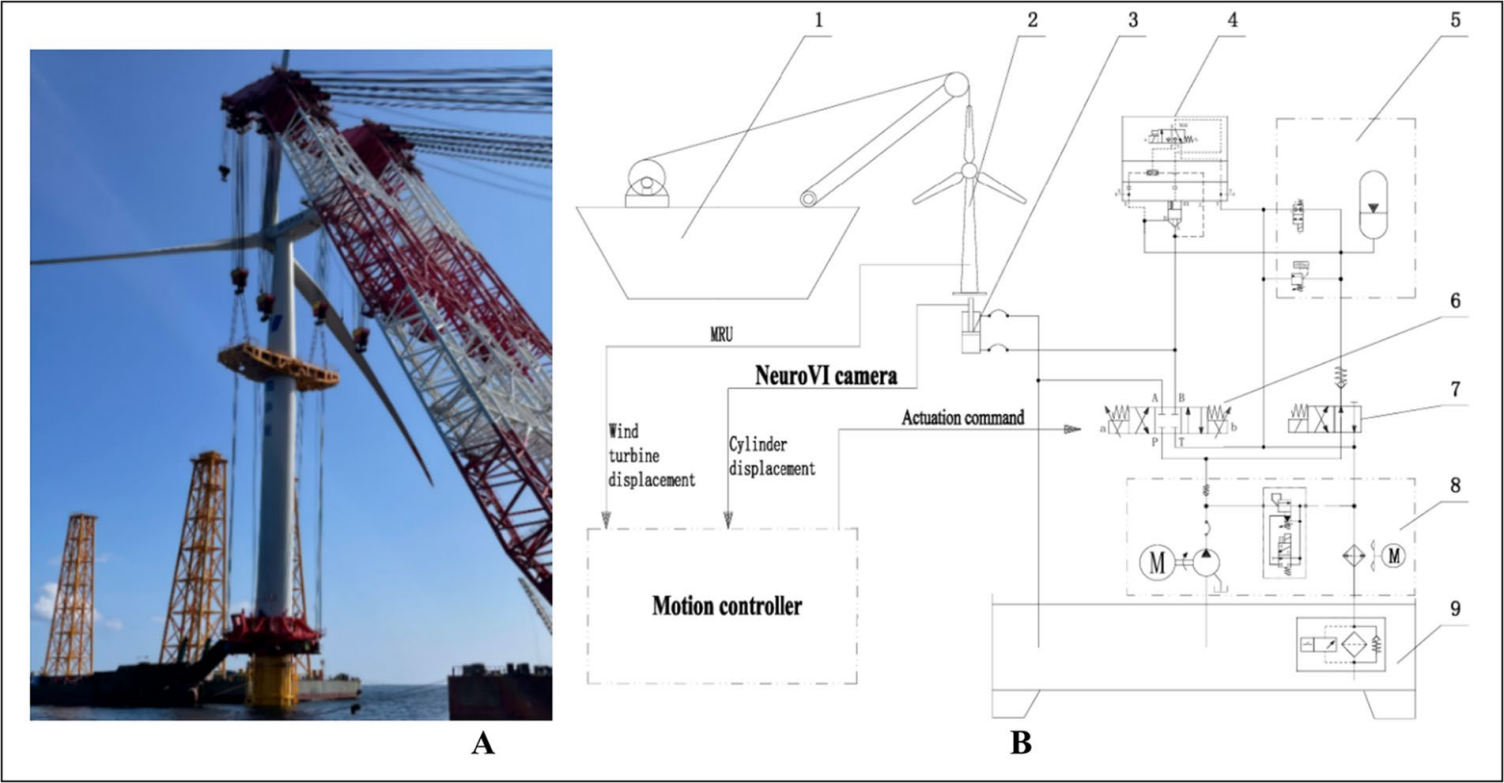
Wave compensation system for integrated installation of offshore wind turbine based on NeuroVI camera. **(A)** Offshore wind turbine. **(B)** The hydraulic system. 1-Crane vessel, 2-Offshore wind turbine, 3-Hydraulic cylinder, 4-Cartridge valve, 5-Accumulator unit, 6-Proportional control valve, 7-Accumulator switch valve, 8-Hydraulic power unit, 9-Oil reservoir.

As illustrated in Figure 1, the proposed compensation system operates on a closed-loop control strategy: A NeuroVI camera monitors hydraulic cylinder displacement in real time, while a high-precision motion reference unit (MRU) tracks the turbine’s position and orientation (Chen et al., 2013). The relative displacement data from both sensors are fed back to the control system. The controller generates drive signals to regulate proportional valves, synchronizing the cylinder’s extension/retraction with the turbine’s heave motion. This system achieves active heave compensation, significantly improving installation stability. In the initial state, the hydraulic power unit (8) is activated, directing hydraulic oil into the accumulator unit (5) via the accumulator switch valve (7). During upward motion of the wind turbine, the cartridge valve (4) opens, while the control unit engages the left position of the proportional control valve (6). This allows rapid oil flow into the cylinder’s rodless chamber, extending the piston. During downward motion, the control unit switches the proportional control valve (6) to its right position, directing oil into the rod-end chamber, thereby retracting the cylinder.

Regarding heave compensation during integrated installation, Zhang Liangjuan et al. improved the design of the throttle orifice in the traditional compensation system, using accumulators to absorb impact energy to achieve vibration reduction, but this method is still in the experimental verification stage (Zhang et al., 2010). Wu Jianzhong’s team proposed a bottom hydraulic cylinder compensation solution for 3 MW turbines, developing a hoisting system with precise positioning and cushioned landing capabilities (Wu et al., 2010). The Third Harbour Engineering Bureau implemented an FIS buffer system in 4 MW installations, achieving multi-system cooperative control (Lu, 2020; Lei and Pan, 2018; Liu et al., 2020). The Kriegers Flak offshore wind farm successfully demonstrated integrated installation of 8.4 MW turbines in shallow waters. However, in deep-sea areas, effective solutions for wave interference in the integrated installation of wind turbines, especially high-frequency dynamic control under strong wave disturbances, are still technically immature (Maria and Jaakko, 2021).

Existing compensation systems predominantly rely on traditional magnetostrictive displacement sensors (Liu et al., 2025). Their contact-based installation is susceptible to mechanical vibrations and oil contamination in harsh sea conditions, resulting in response lag, weak anti-interference capability, and inaccurate high-frequency displacement/velocity feedback. Event cameras (Zhang Y. et al., 2024; Cao et al., 2025) as novel bio-inspired vision sensors, feature microsecond latency and high dynamic range. Their pixels asynchronously respond to luminance changes, generating sparse event streams only for object motion. This enables detection of subtle piston rod displacements. By extracting motion normal vector fields from event point clouds and incorporating cylinder structural parameters, millimeter-level displacement measurement is achievable. Velocity data can be derived from event density variation rates, eliminating quantization delays inherent to traditional sampling while enhancing feedback accuracy and system response, especially under low-light and high vibration conditions.

**Figure 2:**
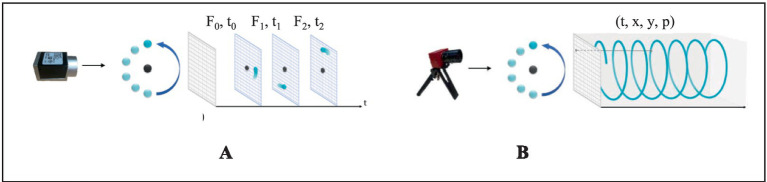
Imaging principles of RGB camera and NeuroVI camera (Chen et al., 2022). **(A)** The frame-based camera captured the RGB pixels with a fixed frequency. **(B)** The NeuroVI camera captured the pixel changement with low latency (Liu X. et al., 2022; Liu and Yang, 2022).

Conventional RGB cameras are prone to motion blur due to limitations in global shutter mechanisms and frame buffering (typically 200 ms/frame), which can lead to loss of critical information in high-speed motion scenarios. In contrast, the core principle of dynamic vision lies in spatiotemporal continuity perception, achieved through bio-inspired vision sensors. In this paradigm, each pixel independently monitors the rate of luminance change, selectively activating only those pixels where brightness variations occur while skipping static backgrounds. When the change exceeds a predefined threshold, the sensor outputs an asynchronous event stream, with each event encoded as a (*x, y, t, p*) quadruple representing pixel coordinates, timestamp, and polarity. Figure 2 illustrates this mechanism. As a young technology sensor, the NeuroVI cameras encounter a significant challenge of insufficient datasets, which impedes the full-fledged advancement of the sensor. Liu et al. recorded a segment of pedestrian behavior to capture body’s movement center (Liu et al., 2020; Liu and Chen, 2020; Tu et al., 2025). Mueggler et al. released a dataset for pedestrian-falling detection, primarily used for human safety early warning (Mueggler et al., 2017). Chen et al. recorded a dataset of highway vehicles, and event points’ segmentation was achieved by a clustering method (Chen et al., 2018). To identify moving objects and improve positional calculations in dynamic scenes, Liu et al. developed a NeuroVI dataset to recognize and deduct the moving vehicles, pedestrians, and bicycles (Liu X. et al., 2022). Despite the availability of several datasets currently, there remains a scarcity of datasets specifically tailored for cylinder displacement under low-illumination scenarios. A comparative analysis of the performance characteristics of these measurement methods is presented in Table 1.

**Table 1:** Performance comparison of displacement measurement methods.

Categorization	LVDT	Magnetostrictive	RGB	NeuroVI
Actual picture	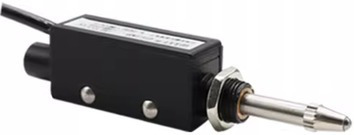	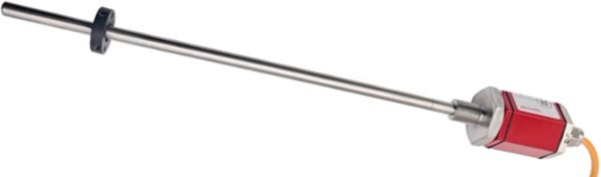	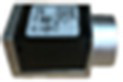	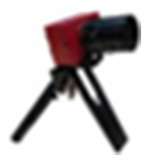
Working principles	Electromagnetic induction	Magnetostrictive effect and time-difference measurement	Physical marker tracking	AI-Enhanced visual analysis
Advantages	Robust performance	Long measurement range (0–8 m)	Cost-Effectiveness	Marker-Free operation
Disadvantages	Range limitations in certain configurations (<1 m)	Fluid contamination sensitivity	Latency constraints	Premium variant costs

Although NeuroVI has been initially applied in multiple fields, its application in hydraulic systems is currently limited. There is a lack of dedicated datasets specifically for detecting cylinder displacement, especially in the aspects of identifying and tracking targets under low-light conditions.

This study synthesizes multiple integrated installation cases to propose a non-contact hydraulic cylinder displacement detection and wave compensation solution using NeuroVI cameras. Our work is the first to apply NeuroVI camera to the field of integrated installation of offshore wind turbine. In summary, our contributions include the following three aspects:

- A spike neural network (SNN) method is designed to filter NeuroVI images, and the sift feature points are leveraged to select the optimal image. A learning network is designed for improving inference speed, which is employed to detect and calculate motion.
- The system employs a NeuroVI camera to achieve non-contact, high-precision, and low-latency displacement detection of hydraulic cylinders, overcoming the limitations of traditional magnetostrictive displacement sensors, which exhibit slow response and susceptibility to interference in harsh marine conditions.
- Through AMESim-Simulink co-simulation and laboratory tests, we systematically validated the system’s response under various disturbance signals. Successful precision docking of a 5.2 MW offshore turbine with its foundation demonstrated the solution’s engineering feasibility and robustness in deep-sea environments.

## 2 Materials and methods

This study proposes an optimized installation process incorporating wave compensation technology based on the 5.2 MW offshore wind turbine. In section 2.1, the NeuroVI camera for hydraulic cylinder displacement detection was detailed introduced. In section 2.2, the signal acquisition performance of traditional displacement sensors and the NeuroVI camera is evaluated in terms of their impact on compensation effectiveness. The results demonstrate the superior efficacy of the proposed approach.

### 2.1 Low-latency NeuroVI camera-based hydraulic cylinder displacement measurement

Despite the success of RGB images in segmentation under good visual illumination, they exhibit subpar segmentation performance in adverse weather conditions, especially in low-illumination scenes where key features are not clear enough. RGB images contain rich information, but in the low-illumination conditions, the image clarity is often compromised. On the other hand, event cameras have the advantage of displaying object contours, but lack detailed information.

Due to the complex maritime environment and camera circuits, event cameras exhibit noise event points in challenging illumination conditions. Moreover, the clarity of NeuroVI images can vary depending on the movement of cylinders. To overcome these challenges, we propose a three stages’ filtering method, aiming at noise reduction, the selection of the best NeuroVI image and movement measurement.

**Figure 3:**
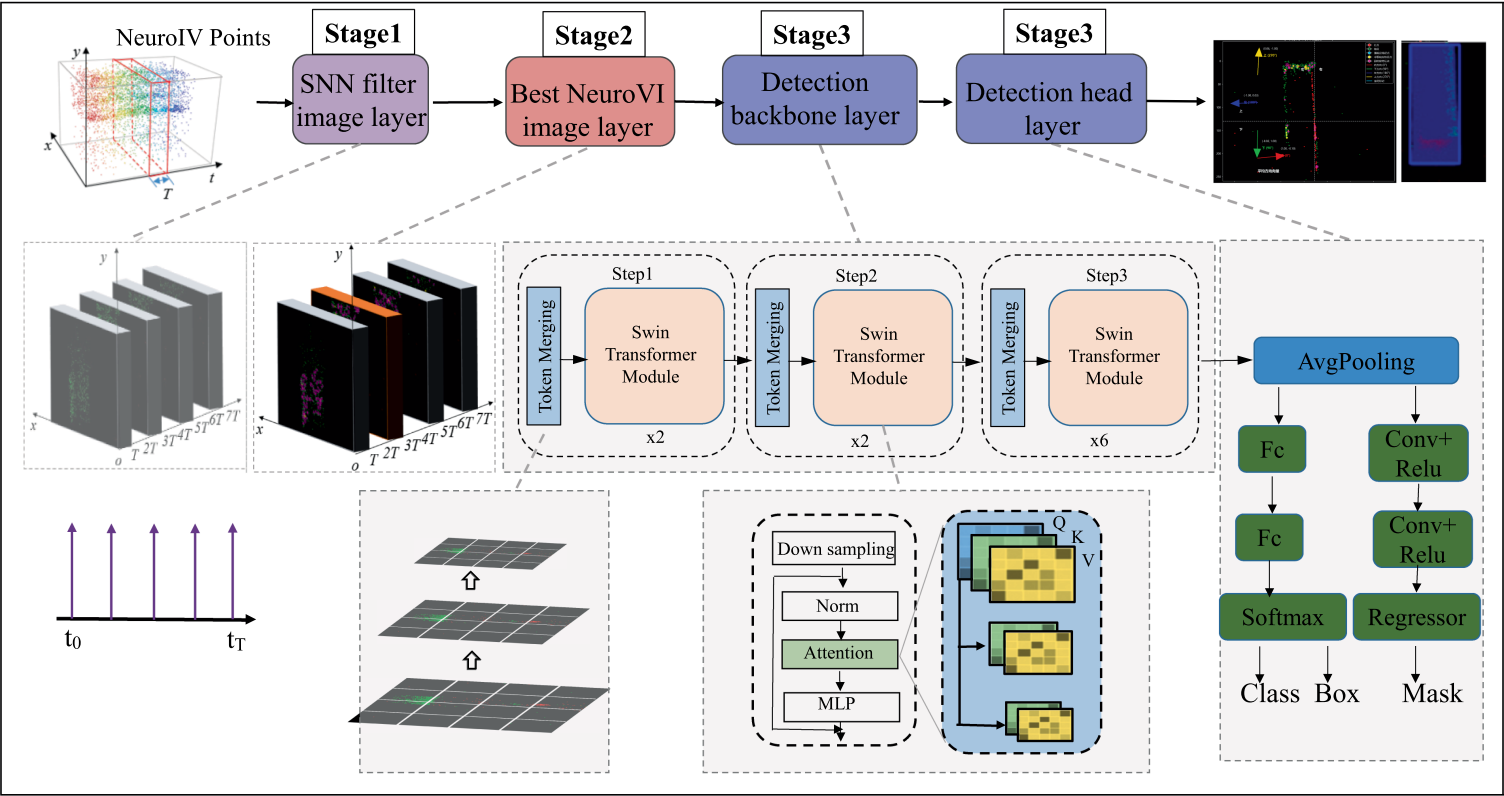
Low-latency NeuroVI camera-based hydraulic cylinder displacement detection process.

As illustrated in Figure 3, this network processing pipeline is meticulously designed and comprises three core stages to achieve efficient image analysis and motion understanding. Stage 1 focuses on data purification. It employs the SNN filter algorithm for intelligent filtering. This algorithm effectively identifies and removes isolated points lacking sufficient similar neighbors within their local neighborhoods. Consequently, it significantly eliminates disruptive discrete noise while maximally preserving and highlighting truly discriminative salient spatial feature points. Stage 2 concentrates on feature matching and view selection. Based on the purified set of key feature points obtained from Stage 1, the system extracts descriptors for these points and performs precise feature matching among temporally or spatially adjacent image frames. This stage intelligently evaluates and selects the single “optimal image” that best aligns with the current analysis target and contains the richest information. Stage 3 constitutes the measurement phase. It applies a deep learning model to the selected optimal image. Employing techniques like model compression, this stage reduces computational complexity and substantially increases inference speed. Ultimately, it precisely calculates the target object’s specific position (coordinates) within the scene and its current direction of motion vector.

#### 2.1.1 Spiking neural networks filter

The event camera converts brightness changes into event points through digital circuits. Event points are marked as a tuple of four elements, *e* = (*x, y, t, p*). Among them, (*x, y*) represents the coordinates of an event point, *t* represents the event time, and *p* represents the event polarity.

**Figure 4:**
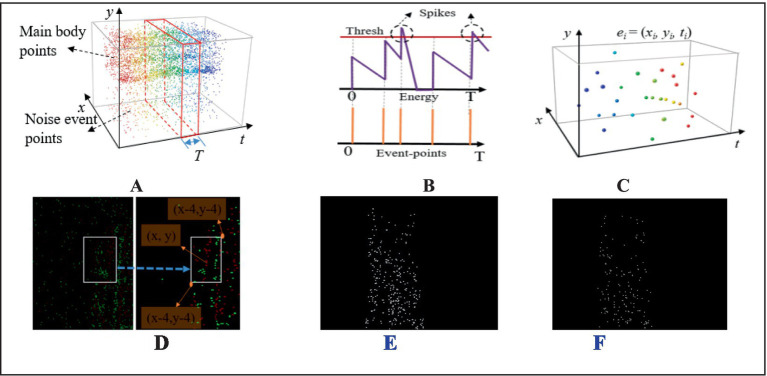
The SNN filtering method of NeuroVI’s image. **(A)** The original event and noise points within a fixed clustering period T. **(B)** The changing trend of potential energy triggered by event points. **(C)** The SNN filtering’s results. **(D)** The ei points are projected on the plan, which are then accumulated within a scope. **(E)** SNN-region method. **(F)** SNN method.

As shown in Figure 4A, the external environment can introduce some sparse noise points through the event camera, but the object’s event points own the advantage of higher density and continuity. Inspired by biological stimulation of neurons, the SNN filter is utilized to remove the aforementioned low-frequency noise. The SNN filter have an event-triggered computation characteristic, and the consecutive excitation of event points will lead to the accumulation of potential energy (Figure 4B). Therefore, the potential energy integration and threshold approach of SNN method can significantly enhance the object features and filter out noisy event points.

In equation 1, $P_{\left(x,y\right)}^{t}P_{\left(x,y\right)}^{t}$ represents the potential energy of a certain pixel at time *t*, and *P_0_* represents the initial potential energy. Every event point will trigger and increase the potential energy with an increment of *ΔP* unit, but $P_{\left(x,y\right)}^{t}P_{\left(x,y\right)}^{t}$ will also dissipate with a fixed decay factor of *γ* at the same time.

(1) $$P_{\left(x,y\right)}^{t}=P_{0}+\sum_{i=0}^{n}(1-\frac{1}{T}\int_{t(i)}^{T}\mathrm{\gamma dt}).\mathrm{\Delta P}$$

Among them, *n* represents the total number of event points, *T* represents the clustering interval, and *t*(*i*) represents the time when the *i-th* event point occurred within the interval *T*. In equation 2, *θ* represents the threshold of potential energy, which is determined by experiment. When *P^t^* surpasses the threshold *θ*, the filtered events will be triggered and recorded. Each filtered event point can be simplified as a 3D spatial distribution point with coordinates (*x_i_*, *y_i_*, *t_i_*), as illustrated in Figure 4C.

(2) $$\varepsilon =\{\{\varepsilon_{\left(x,y\right)}^{j},(j=1,2\cdots \cdots ,m)\}|P_{\left(x,y\right)}^{t}>\theta \}$$

The filtering operation not only eliminates noise points, but also weakens the edge information of key features. Considering that there are many feature points near the key features, we calculate the sum of *ei* points on the projection plan to highlight edge features (Figure 4D). The cumulative range is distributed within in ([x − 4, y − 4], [x + 4, y + 4]). The relationship between the events number and pixel values is presented in equation 3. As $0<(\frac{1}{1+e^{-M}}-0.5)<0.5$ , the pixel value of *δ*(*M*) can be constrained within the scope of [0, 255].

(3) $$\left\{ \begin{array}{c} M=\int_{x-4}^{x+4}\int_{y-4}^{y+4}\varepsilon_{\left(x,y\right)}\text{dxdy}\\ \delta (M)=255*2*(\frac{1}{1+e^{-M}}-0.5) \end{array}\right.$$

Several existing studies (Chen et al., 2022) employ the Shared Nearest Neighbor (SNN) method. In contrast to their approaches, the SNN-region method defined in Equation 3 was adopted. Following the SNN processing, the cumulative range distribution is constrained within the region ([x − 4, y − 4], [x + 4, y + 4]). Through this method, we enhance the color contrast in salient feature regions. (Figures 4E,F).

#### 2.1.2 Selection of the best NeuroVI image

**Figure 5:**
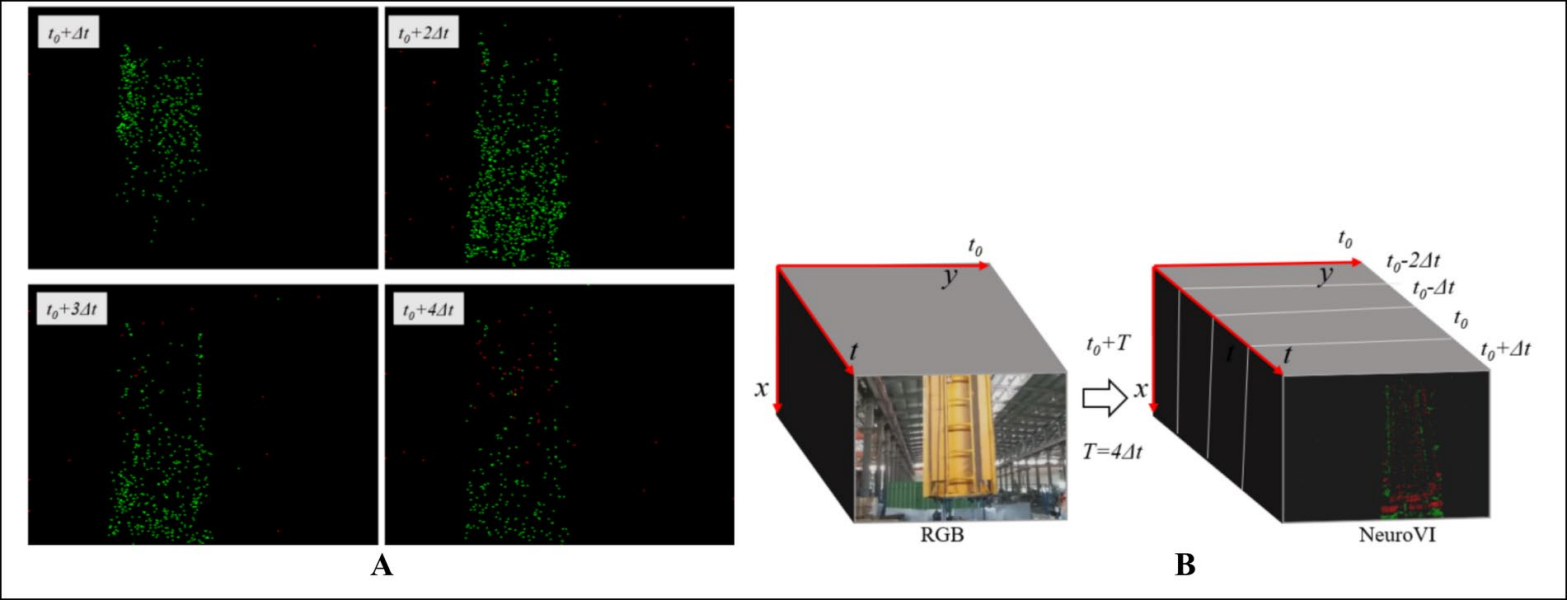
The characteristic embedding method for selecting the best SNN-filtered image. **(A)** Due to cylinder movement, the adjacent images of same scene own different clearances. **(B)** One RGB image matches four NeuroVI images.

An image with stable and significant features is a prerequisite for ensuring detection accuracy. Influenced by the cylinder speed, sea conditions, and irregular surface appearance, the extracted NeuroVI images appear unstable (Figure 5A). Increasing the clustering time interval will extends the features, while reducing the clustering time interval will weaken the features. Besides, the feature positions among adjacent images are nearly identical, so there is no need to worry about positional drift. Therefore, it’s necessary to select the optimal image under a fixed sampling period.

We extract four consecutive images: *t* − *2Δt*, *t* − *Δt*, *t*, and *t + Δt*, which are represented as slices in Figure 5B. From these four consecutive image slices, our goal is to select the most suitable NeuroVI image that exhibits the most obvious features. Obviously, images with the longest contour line display the most distinct features. Since the number of sift feature points is directly proportional to the length of the contour line, we evaluate and select the best image by calculating the number of sift feature points.

The convolution operation between the image and the Gaussian function can blur images to different degrees. Therefore, a blurred image can be defined as *L*(*x, y, σ*) function in Equation 4. Among them, *G*(*x, y, σ*) is a scale-changed Gaussian convolution kernel, and *I*(*x, y*) is the original image. (*x, y*) denotes the pixel coordinates, and *σ* can influence the blurriness degree of the image.

(4) $$\left\{ \begin{array}{c} L(x,y,\sigma )=G(x,y,\sigma )*I(x,y)\\ G(x,y,\sigma )=\frac{1}{2\pi \sigma^{2}}e^{\frac{-(x^{2}+y^{2})}{2\sigma^{2}}} \end{array}\right.$$

The convolution calculation with two different σ value can blur image with different degrees. Subsequently, the subtraction operation is applied between the two adjacent blurred images (equation 5), resulting in another image, namely the Difference of Gaussians (DOG). To find the local maximum and minimum values, a certain pixel in the DOG needs to be compared with its adjacent pixels. If a pixel owns the highest or lowest value among its neighboring pixels, it is defined as an extreme point in the original image.

(5) $$\begin{array}{c} D(x,y,\sigma )=(G(x,y,\mathrm{k\sigma})-G(x,y,\sigma ))*I(x,y)\\ \;\;\;\;\;\;\;=L(x,y,\mathrm{k\sigma})-L(x,y,\sigma ) \end{array}$$

Generally, an ideal extreme point exhibits smaller curvature along the edge and larger curvature in the direction perpendicular to the edge. The application of the DOG operator tends to produce multiple extreme points, some of which may be unstable and required to be eliminated. To achieve this, the Hessian matrix is calculated at each extreme point. The curvature is then derived by evaluating the 2 × 2 Hessian matrix *H*, as described in equation 6.

(6) $$H=[\begin{array}{cc} D_{\mathrm{xx}} & D_{\mathrm{xy}}\\ D_{\mathrm{xy}} & D_{\mathrm{yy}} \end{array}]$$

At the extreme points, the values of *D_xx_*, *D_xy_*, and *D_yy_* are computed by using the difference method. *α* denotes the maximum eigenvalue of the matrix, and *β* denotes the minimum eigenvalue of the matrix. *Tr*(*H*) denotes the trace of matrix *H*, and *Det*(*H*) denotes the determinant of matrix *H*. The relevant calculation relationship is illustrated in Equation 7.

(7) $$\left\{ \begin{array}{c} \mathrm{Tr}(H)=D_{\mathrm{xx}}+D_{\mathrm{yy}}=\alpha +\beta \\ \det(H)=D_{\mathrm{xx}}+D_{\mathrm{yy}}-D_{\mathrm{xy}}^{2}=\alpha .\beta \end{array}\right.$$

*γ = α/β* represents the ratio of the two eigenvalues. The relationship among the trace, determinant and *γ* is calculated in equation 8.

(8) $$\frac{\mathrm{Tr}{\left(H\right)}^{2}}{\det(H)}=\frac{{\left(\alpha +\beta \right)}^{2}}{\alpha \beta }=\frac{{\left(\gamma \beta +\beta \right)}^{2}}{\gamma \beta^{2}}=\frac{{\left(\gamma +1\right)}^{2}}{\gamma }$$

**Figure 6:**
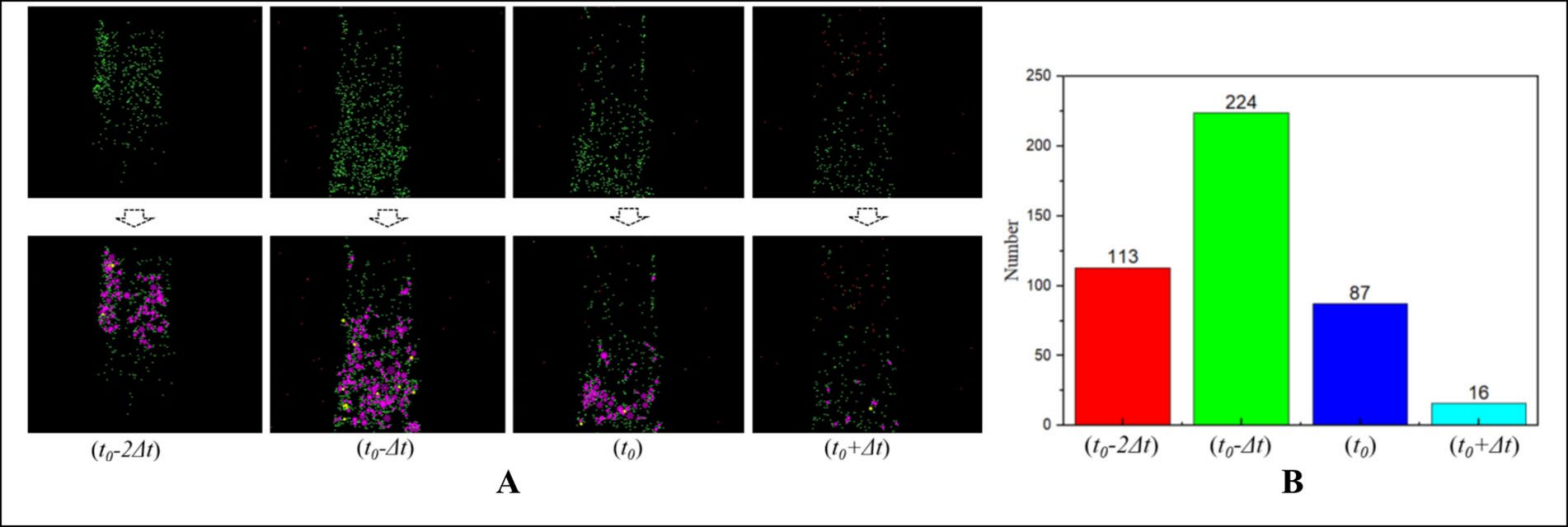
The selection of best NeuroVI image based on the number of sift feature points. **(A)** The several adjacent image slices from the same scenario. **(B)** The second image yields the highest number of sift feature points, making it the optimal choice among all the images in the scenario.

The reliability of extreme points is associated with the *γ* value. Usually, a higher *γ* value indicates smaller curvature along the edge and larger curvature in the direction perpendicular to the edge. To filter out extreme noise points, a threshold is set by using equation 9, and the extreme points with values greater than this threshold are considered reliable. Through experimental evaluation, a threshold value of *Tr* = 12.1 is finally determined. One example of selection process in a scenario is demonstrated in Figure 6.

(9) $$\frac{\mathrm{Tr}{\left(H\right)}^{2}}{\det(H)}>\frac{{\left(r+1\right)}^{2}}{r}=12.1$$

#### 2.1.3 Dataset construction and movement measurement

**Figure 7:**
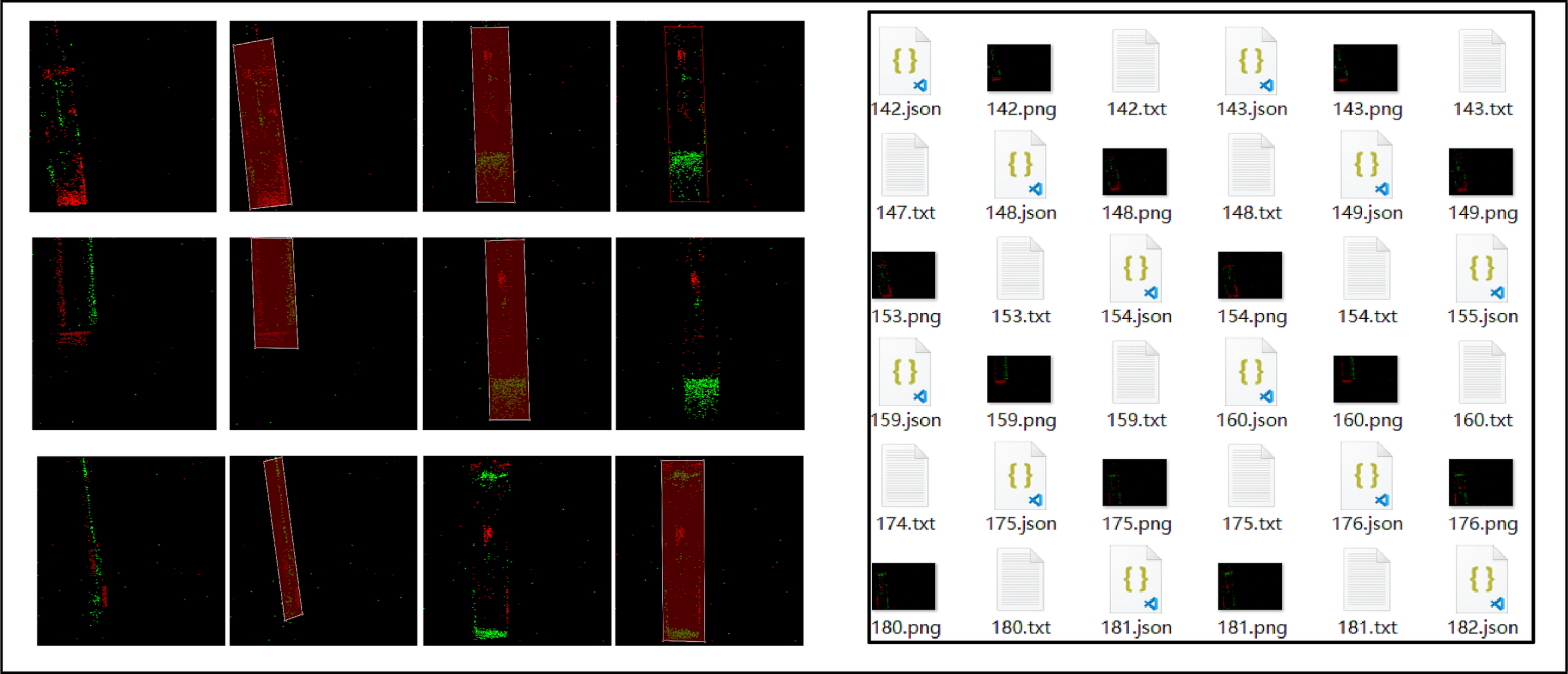
Dataset construction.

At the edge positioning point of the hydraulic cylinder motion mechanism, the moving guide rod is rigidly fixed to the motion plate using high-strength bolts, forming a high-stiffness kinematic pair. The motion process was captured with μs-level temporal precision using a DAVIS346 event camera, generating asynchronous event streams. Subsequently, we employed a SNN filter algorithm to extract images of the moving edge rod, performed precise image annotation, and constructed a specialized dataset. The resulting dataset is illustrated in Figure 7.

After extracting the optimal image, we designed a deep learning detection network (Figure 3). Given the superior inference capability of the Transformer architecture, we adopted it for object and motion detection. The Transformer structure incorporates three feature attention matrixes, namely the Query/Key/Value (Q/K/V) matrices. To reduce the computational overhead of matrix operations during inference, the three layers share the query matrix from the first Transformer layer. Additionally, the query matrix of the first Transformer layer is downscaled while maintaining its original dimensions. Since this approach only detects moving objects, it significantly enhances detection reliability.

**Figure 8:**
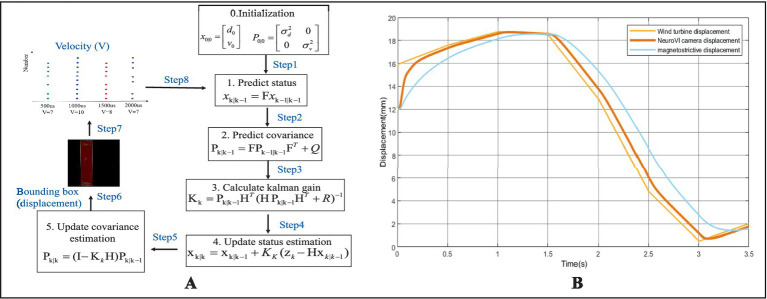
Real-time position detection with event camera image, velocity prediction, and Kalman filtering methods. **(A)** The Kalman filtering method. **(B)** Performance metrics.

Event cameras detect moving objects with microsecond (μs) latency, whereas neural network-based object detection incurs 7–8 millisecond (ms) delays. To enhance real-time processing performance, current event density (spatiotemporal thickness) provides real-time velocity input, and previously estimated object position serves as positional input. Defining the state vector at time k as xk = [dk, vk]T, we design the Kalman filter with state equation 10, measurement equation 11 and covariance matrices equation 12. Δt denote the temporal interval between event-based detections. σa denotes the standard deviation of acceleration noise. σb denotes the standard deviation of displacement noise. F denotes the state transition matrix. H denotes the observation matrix. The Kalman filtering method is shown in Figure 8A. Through experiment, the performance metrics are shown in Figure 8B.

(10) $$\left\{ \begin{array}{c} x_{k|k-1}=Fx_{k-1|k-1}\\ F=[\begin{array}{cc} 1 & \mathrm{\Delta t}\\ 0 & 1 \end{array}] \end{array}\right.$$

(11) $$\left\{ \begin{array}{c} z_{k}={\mathrm{Hx}}_{k}+v_{k}\\ H=[1\;\;0] \end{array}\right.$$

(12) $$Q=[\begin{array}{cc} \frac{\Delta t^{4}}{4}\sigma_{a}^{2} & \frac{\Delta t^{3}}{2}\sigma_{a}^{2}\\ \frac{\Delta t^{3}}{2}\sigma_{a}^{2} & \Delta t^{2}\sigma_{a}^{2} \end{array}],R=\sigma_{z}^{2}$$

### 2.2 AMESim-Simulink co-simulation framework for wave compensation systems

To verify the effectiveness of the wave compensation system based on NeuroVI and provide theoretical support for experimental testing, this study employs AMESim-Simulink co-simulation to analyze dynamic compensation performance under different sensor configurations. It will provide an effective basis for control parameter optimization and hardware test configuration (Zhou, 2023).

#### 2.2.1 Simulation model construction for wave compensation system

1) Hydraulic system modeling in AMESim

**Figure 9:**
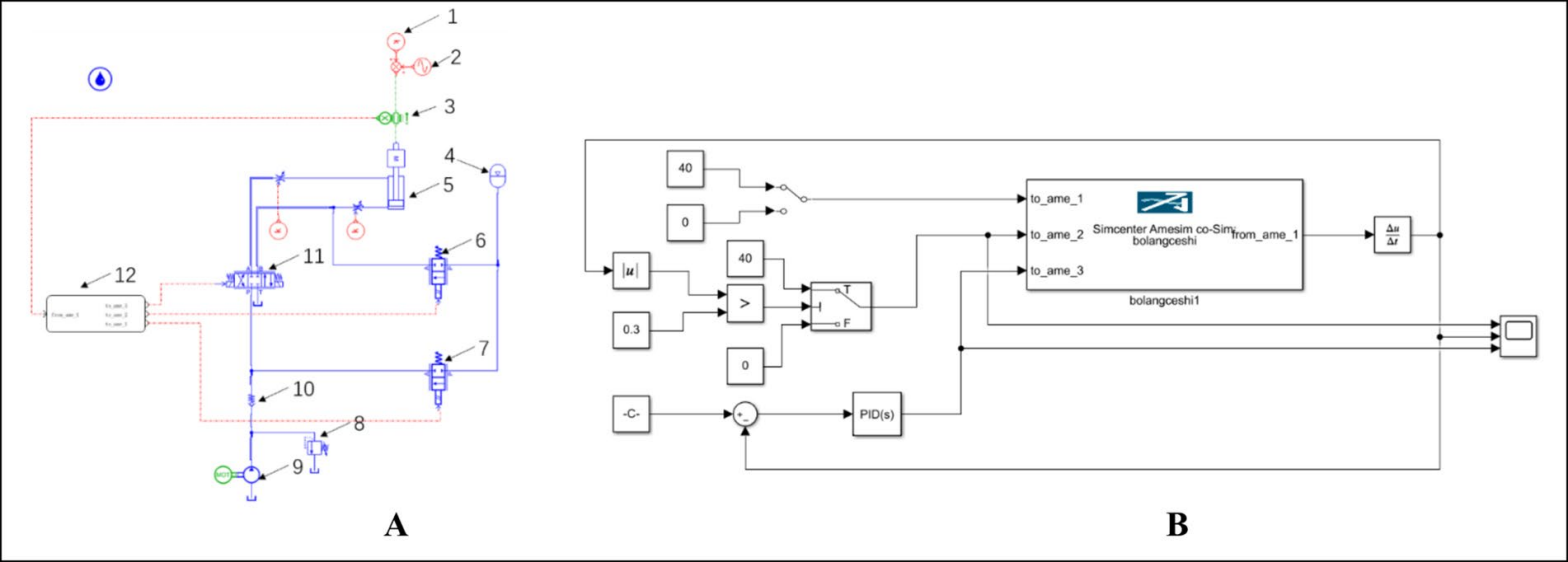
Simulation model configuration. **(A)** Hydraulic system model in AMESim environment. **(B)** Control system model in Simulink environment. 1-Wind turbine pressure, 2-Wind turbine displacement, 3-Cylinder displacement signal, 4-Accumulator, 5-Compensation cylinder, 6-Cartridge valve, 7-On/off valve, 8-Relief valve, 9-Hydraulic pump, 10-Check valve, 11-Proportional directional valve (electromagnetic), 12-Simulink module.

When constructing the hydraulic system model in AMESim, components from different libraries are color-coded for clarity: blue represents hydraulic library elements, red denotes signal library components, and green indicates mechanical library parts. The controller is implemented using the Simulink interface module from the *Interface* library. The AMESim model of the hydraulic system is illustrated in Figure 9A.

2) Control system modeling in MATLAB/Simulink

Based on the operational principles of wave compensation systems for integrated installation of offshore wind turbine, the control model regulates cylinder displacement by adjusting the proportional directional valve opening. During cylinder extension, the pump and accumulator rapidly replenish hydraulic fluid to maintain flow demand. Conversely, during cylinder retraction, the accumulator absorbs excess fluid to mitigate hydraulic shock. The Simulink implementation of this control system is presented in Figure 9B.

3) Dynamic modeling of wave compensation systems

Heave force analysis and modeling assumptions for integrated installation of offshore wind turbine (Lei, 2015; Zhang and Sheng, 2022):

Assumption 1: The hook, rigging, and turbine share colinear centers of mass along the heave axis, forming a unified rigid body without relative motion.

Assumption 2: Secondary effects are neglected, such as aerodynamic drag forces, self-weight and elastic deformation of lifting assemblies, and friction between compensation cylinders and turbine base.

Simplification basis for wave compensation system dynamics model:

Simplification 1: The vessel’s heave motion induced by waves is idealized as a sinusoidal excitation.

Simplification 2: During turbine lowering, the cables are treated as rigid bodies, and the hook moves downward at a constant velocity (*v*).

Simplification 3: The wave compensation mechanism is installed on the pile-founded platform, with its key features including mounting atop the pre-installed foundation piles. The compensation system is modeled as a variable spring-damper with equivalent stiffness (*k*), damping coefficient (*c*), and turbine displacement (*x*).

**Figure 10:**
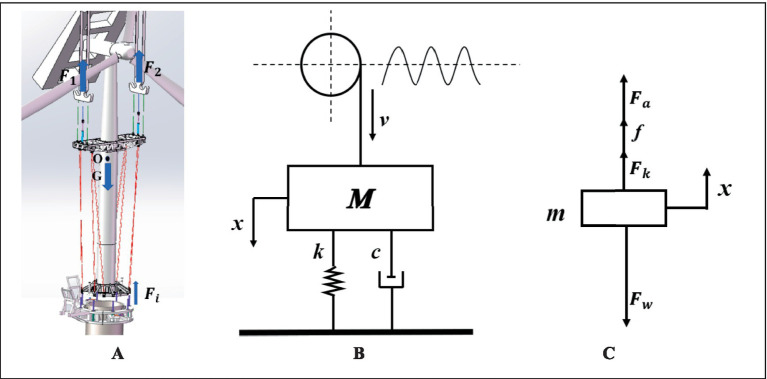
Dynamic modeling schematics for integrated wind turbine hoisting system. **(A)** Force analysis diagram. **(B)** Compensation system dynamics model. **(C)** Piston force analysis diagram.

Based on the preceding assumptions and simplifications, the mechanical analysis of the wind turbine hoisting system and the dynamic model of the compensation system are presented in Figures 10A,B.

Based on the established assumptions and dynamic model, the force balance equation for the system’s center of mass is derived as follows (Jia et al., 2017; Niu et al., 2017):

When the turbine descends at constant velocity (*a = 0*):

(13) $$F_{1}+F_{2}+(-G)+\sum_{i=1}^{8}F_{i}=0$$

Wave-induced acceleration (*a ≠ 0*) modifies the balance to:

(14) $$\mathrm{Ma}=\mathrm{Mg}-[F_{1}+F_{2}]-\sum_{i=1}^{8}F_{i}$$

In equations 13, 14 $F_{1}$ , $F_{2}$ represents the tension forces from crane hooks, $F_{i}(i=1,2,3,4,5,6,7,8)$ represents the thrust forces from 8 compensation cylinders, *G = Mg* represents total weight of turbine and rigging, where *M* represents mass.

When the turbine contacts the compensation cylinders, the piston becomes the critical load-bearing component (Liu, 2010; Yao, 2015). The force analysis diagram is shown in Figure 10C. The external load acting on the piston rod is derived as shown in equation 15:

(15) $$F_{w}=\mathrm{Mg}-F_{1}-F_{2}$$

The piston rod and its coupled load exhibit dynamic response shown in equation 16:

(16) $$F_{a}=m\frac{d^{2}x}{dt^{2}}$$

The liquid in the cylinder acts as an equivalent spring. When compressed or stretched, this spring-like medium deforms, with the deformation equal to the piston rod’s displacement within the hydraulic cylinder. The compressed gas in the system tends to restore its original state, generating a restoring force that can be expressed as shown in equation 17:

(17) $$F_{k}=\mathrm{kx}$$

The liquid in the cylinder generates resistance during motion. This resistance force magnitude depends on three key factors: the hydraulic oil flow rate, the oil viscosity, and the resistance coefficient. Their relationship can be expressed as shown in equation 18:

(18) $$f=c\cdot \frac{\mathrm{dx}}{\mathrm{dt}}$$

In summary, the equation of motion for the hydraulic cylinder piston can be derived as shown in equation 19:

(19) $$m\frac{d^{2}x}{\mathrm{dt}2}+\mathrm{kx}+c\cdot \frac{\mathrm{dx}}{\mathrm{dt}}=\mathrm{Mg}-F_{1}-F_{2}$$

#### 2.2.2 Analysis of simulation results

1) Compensation performance simulation of displacement sensor/NeuroVI camera under step excitation

**Figure 11:**
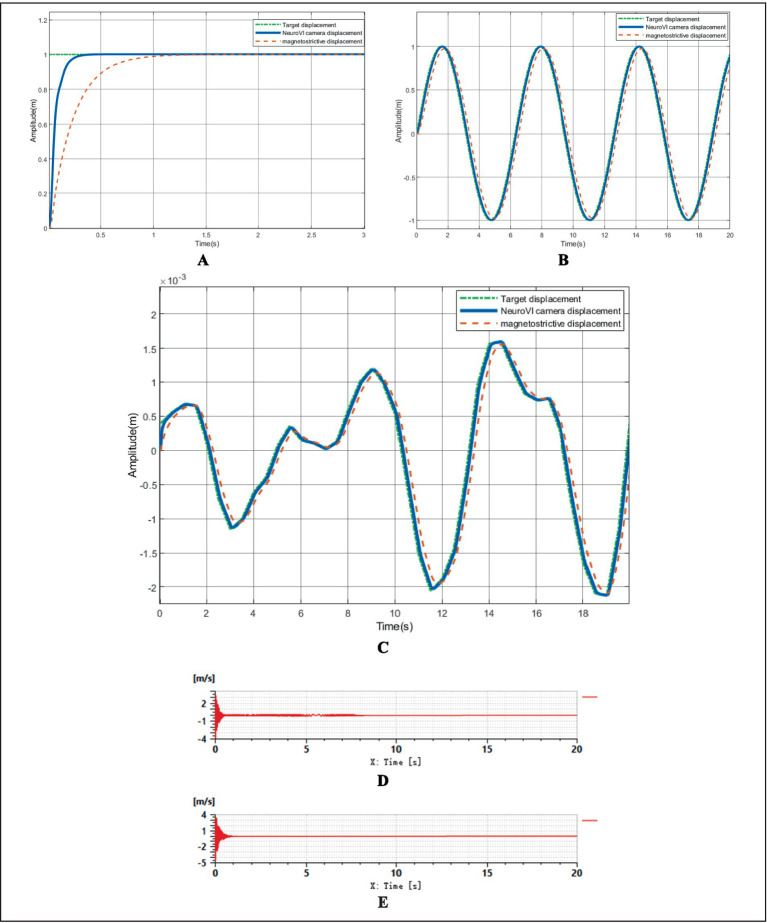
The results of AMESim-Simulink co-simulation. **(A)** Comparative simulation of compensation effects under step signal response. **(B)** Comparative simulation of compensation effects under sinusoidal signal tracking performance. **(C)** Comparative simulation of compensation effects under irregular waves. **(D)** Compensation cylinder velocity under conventional displacement sensor. **(E)** Compensation cylinder velocity under NeuroVI camera-based detection.

The target displacement was configured as a unit step input (amplitude = 1). As shown in Figure 11A, the system maintained stability when using the conventional displacement sensor; however, it exhibited an extended settling time. In comparison, the NeuroVI camera-based system demonstrated significantly improved dynamic response, achieving stable-state regulation within 0.3 s—a marked enhancement in transient performance over traditional sensor methods.

2) Compensation performance simulation of displacement sensor/NeuroVI camera under sinusoidal excitation

The target displacement was modeled as a sinusoidal signal with an amplitude of 1, where the ideal output followed a standard sine function. As illustrated in Figure 11B, the displacement signal acquired by the conventional sensor exhibited a noticeable phase lag compared to the target displacement, indicating a slight delay in response. In contrast, the NeuroVI camera-based displacement tracking demonstrated near-perfect alignment with the reference signal, achieving significantly shorter settling time and higher accuracy. This confirms that the NeuroVI camera ensures superior system stability over traditional displacement sensors.

3) Compensation performance simulation of displacement sensor/NeuroVI camera under irregular waves

Ocean waves exhibit high uncertainty and randomness, typically characterized as irregular waves in hydrodynamics (Su et al., 2021). Through extensive experiments and field data analysis, researchers have established spectral wave models, including the Pierson-Moskowitz (P-M) spectrum, ITTC two-parameter spectrum, and JONSWAP spectrum. Among these, the JONSWAP spectrum provides the closest approximation to real-world wind-wave conditions. Thus, this study employs the JONSWAP spectrum to simulate wave profiles under Sea State 4 (Zhao et al., 2024; Yin and Ran, 2023). Using the generated wave profile as the target displacement, the simulation results are presented in Figure 11C. The data demonstrate that the displacement signals acquired by the NeuroVI camera exhibit the highest consistency with the target displacement, achieving significantly superior compensation performance compared to conventional methods.

4) Stability comparison of compensation performance: displacement sensor vs. NeuroVI camera

To simulate the dynamic load during wind turbine lowering, a ramp signal was applied to represent the gradually increasing force on the compensation cylinder. Meanwhile, wave-induced disturbances were modeled as a sinusoidal signal (Huang et al., 2017), replicating realistic marine conditions during integrated installation of offshore wind turbine.

As demonstrated in Figures 11D,E, the velocity response of the compensation cylinder reveals significant performance differences between signal acquisition methods. The NeuroVI camera-based system exhibits superior stabilization, maintaining near-constant cylinder velocity compared to conventional displacement sensors. These results confirm that the proposed NeuroVI-based wave compensation system ensures a safe and stable operational environment for integrated installation of offshore wind turbine.

## 3 Results

To validate the displacement detection capability of the NeuroVI camera in the wave compensation system, a scaled-down prototype was rigorously tested under laboratory conditions. Subsequently, the system was deployed in a real-world offshore wind farm during integrated installation of offshore wind turbine. The results demonstrate that the proposed wave compensation system based on the NeuroVI camera meets all operational requirements.

### 3.1 Displacement measurement using NeuroVI camera

**Figure 12:**
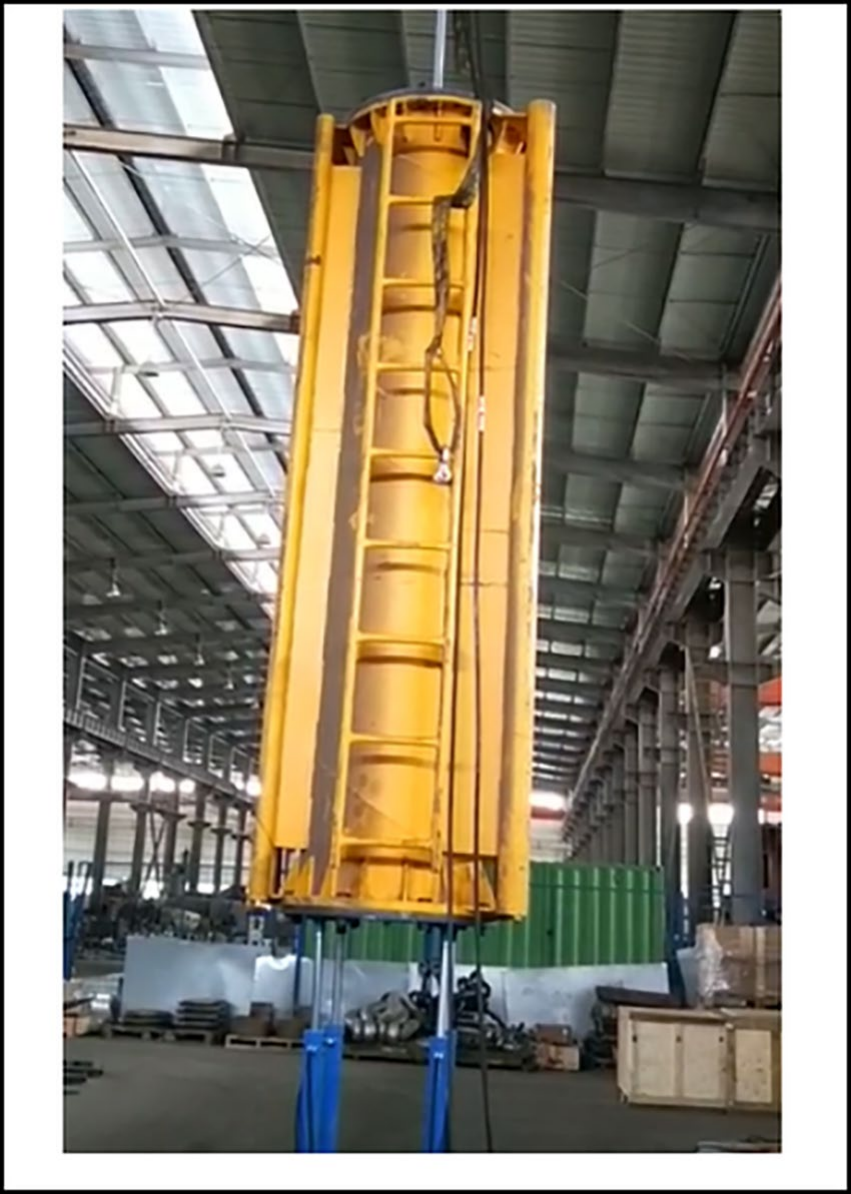
Scaled experimental model of the wave compensation system.

To evaluate the NeuroVI camera’s displacement detection performance, a scaled-down prototype was built in the laboratory. As shown in Figure 12, the setup used a slender counterweight suspended by an overhead crane to simulate wave-induced motions during turbine lifting. Four actively controlled hydraulic cylinders supported the counterweight, synchronizing with its movement to mitigate sudden load impacts.

**Figure 13:**
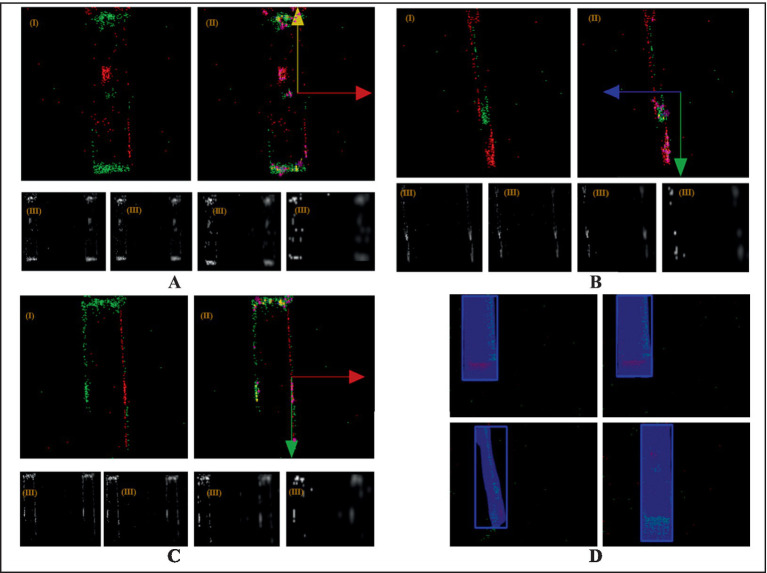
Cylinder motion imaging analysis. **(A)** Upward-right movement. **(B)** Downward-left movement. **(C)** Downward-right movement. **(D)** Displacement measurement via deep learning-based detection (bounding box center point tracking).

During experiments, the hydraulic cylinder displacement was calculated by processing camera images. Based on the polarity of the event points, we accumulate the events and generate an image through image projection. The location corresponding to the maximum positive value of the event points corresponds to red (255), while the location corresponding to the maximum negative value corresponds to green (255). As defined by the direction of motion shown in Figure 13, the object edges facing the direction of motion appear red, whereas the edges opposite to the direction of motion appear green. The color representation in the image and its correspondence to the motion direction are illustrated in Figure 13. Areas where the hydraulic cylinder advances exhibit increased luminance, generating red event points. Consequently, the direction with more red points or fewer green points is the movement direction of the hydraulic cylinder. The displacement of the hydraulic cylinder is calculated by implementing a neural network to identify edge positions in the images, and then determining the cylinder’s position through coordinate transformation from the camera coordinate system to the world coordinate system. The velocity was derived from the average count of red edge points in the image sequence.

The experimental imaging records of different objects are presented in Figure 13, where (I) shows the SIFT-optimized feature-selected images, (II) indicates the hydraulic cylinder’s movement direction, and (III) displays the extracted DoG images during SIFT feature processing. In Figure 13A, the predominant green points in the lower region and red points in the right region indicate upward movement of the object with concurrent rightward motion of the hydraulic cylinder. Figure 13B shows this pattern reversed, where increased red point density in both lower and left regions corresponds to downward object movement and leftward cylinder displacement. Similarly, Figure 13C reveals a configuration with green point accumulation in the upper area and red point predominance on the right side, confirming downward object motion accompanied by rightward cylinder movement.

**Figure 14:**
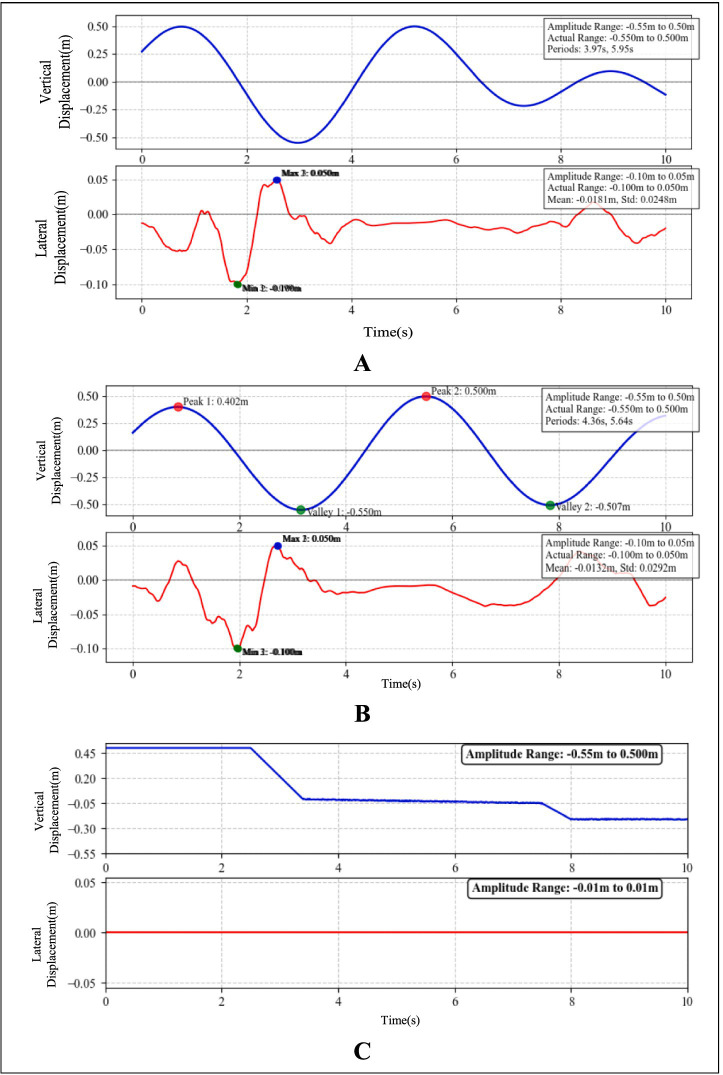
Bidirectional displacement tracking of cylinder motion. **(A)** Heave variable-amplitude with random lateral sway. **(B)** Heave constant-amplitude with random lateral sway. **(C)** Step-response characterization.

The experimental study recorded three characteristic motion patterns of the hydraulic cylinder in Figure 14. In Figure 14A, the first pattern featured heave extension/retraction cycles with amplitude modulation while simultaneously exhibiting random lateral oscillations about the central axis. In Figure 14B, the second motion pattern consisted of constant-amplitude reciprocating movement along the heave axis combined with random lateral oscillations about the central axis. In Figure 14C, the third motion pattern combines stepwise heave displacement with complete lateral stabilization.

### 3.2 Application of NeuroVI camera-based wave compensation systems

**Figure 15:**
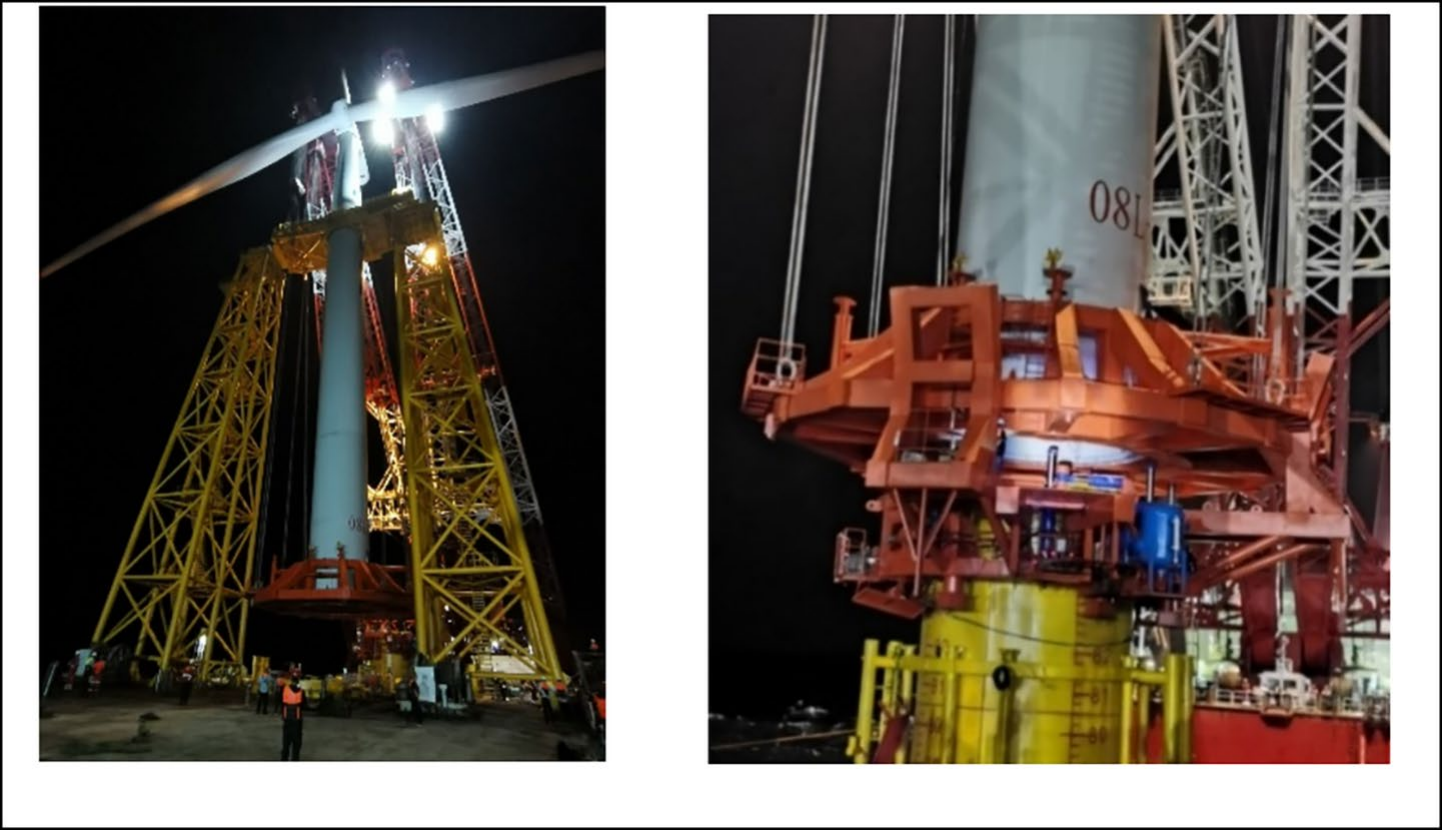
Integrated installation of offshore wind turbine with active wave compensation.

To validate the effectiveness and engineering feasibility of the wave compensation system, field verification was conducted during integrated installation of offshore wind turbine. The newly designed wave compensation system demonstrated significant performance, maintaining synchronized displacement between the hydraulic cylinders and the turbine throughout the operation, enabling stable lowering without any abrupt movements. Compared to segmented installation methods, this approach substantially reduced time requirements, the entire process from preparatory phase to final bolt fastening between turbine and foundation was completed in under 6 h. Figure 15 illustrates key operational phases of both the integrated installation of offshore wind turbine and the wave compensation system.

**Figure 16:**
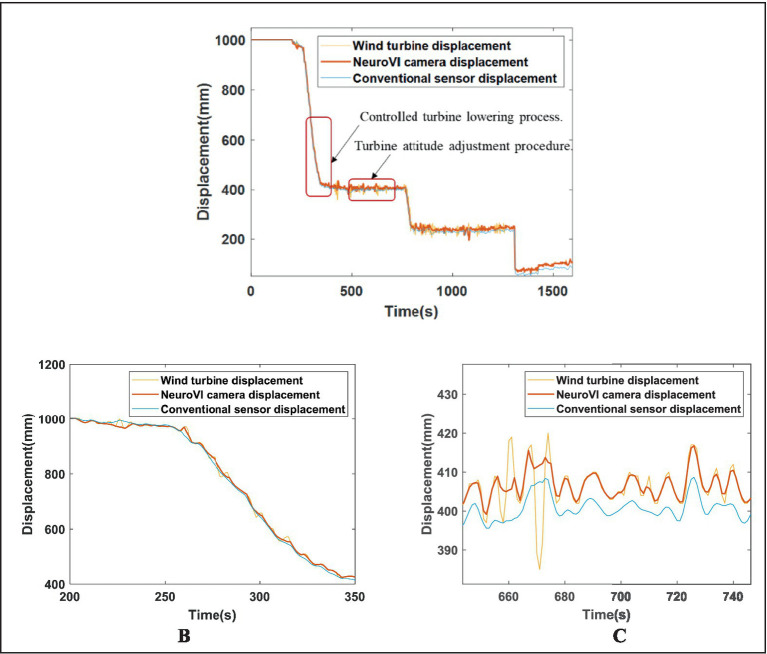
**(A)** Displacement profiles during integrated installation of offshore wind turbine with wave compensation. **(B)** Controlled turbine lowering process. **(C)** Turbine attitude adjustment procedure.

Displacement data of the wind turbine were acquired MRU, while hydraulic cylinder displacements were measured separately using NeuroVI cameras and Conventional displacement sensor, with analytical processing results illustrated in Figure 16A. During the initial 0–300 s phase, the compensation cylinders remained in a fully extended standby configuration awaiting turbine lowering, during which the measured displacement values reached the maximum amplitude due to the absence of external loading forces. During the intermittent periods of 300–750 s, 850–1,250 s, and 1,350–1,600 s, the system entered turbine attitude adjustment phases where the hoisting mechanism maintained a fixed heave position while responding exclusively to wave-induced forces. The displacement oscillated with wave motions. During the remaining operational periods, the system executed the active turbine lowering phase, where the displacement exhibited a near-linear descending trajectory resulting from the superposition of controlled hoisting motion and wave-induced oscillations. To ensure stable turbine installation throughout the process, the wave compensation system operates through coordinated power supply from both hydraulic pumps and accumulators, with the control unit automatically adjusting the opening of proportional valves to drive precise cylinder movements, thereby achieving effective wave motion compensation.

To evaluate the synchronization between cylinder displacement and turbine displacement, an enlarged view of selected intervals is presented in Figures 16B,C. During both the turbine lowering and attitude adjustment phases, the cylinder displacement maintained precise alignment with the turbine displacement while exhibiting near-constant velocity. The NeuroVI camera-based measurements demonstrated superior congruence with actual turbine displacement compared to conventional methods, achieving exceptional synchronization performance. This effective motion coupling confirms the system’s outstanding wave compensation capability, particularly in meeting heave compensation requirements.

## 4 Conclusion

This study developed a novel wave compensation system based on NeuroVI cameras to address the installation challenges of 5.2 MW offshore wind turbines. The system acquires cylinder displacement data through NeuroVI cameras and dynamically adjusts compensation cylinder strokes via control units to achieve displacement balance control during the pile-top foundation and turbine docking phase. Practical results demonstrate:

- High-Precision Displacement Measurement:

The NeuroVI camera, leveraging bio-inspired vision principles, captures edge-event point clouds of moving surfaces in real time. By combining spatiotemporal correlation analysis with cylinder mechanical parameters, it achieves millimeter-level displacement resolution with faster response times than conventional displacement sensors.

- Successful Field Validation:

The system demonstrated reliable performance during the integrated installation of a 5.2 MW turbine under complex marine conditions. The compensation cylinder maintained synchronous displacement with the turbine, effectively eliminating secondary collision risks.

## Data Availability

The original contributions presented in the study are included in the article/supplementary material, further inquiries can be directed to the corresponding author.
